# Factors associated with habitual sleep duration in US adults with hypertension: a cross-sectional study of the 2015–2018 National Health and Nutrition Examination Survey

**DOI:** 10.1186/s12889-021-12465-2

**Published:** 2022-01-06

**Authors:** Everlyne G. Ogugu, Sheryl L. Catz, Janice F. Bell, Christiana Drake, Julie T. Bidwell, James E. Gangwisch

**Affiliations:** 1 Betty Irene Moore School of Nursing, University of California, Davis 2570 48th Street, Sacramento, CA 95817 USA; 2 Department of Statistics, University of California, Davis 399 Crocker Ln, Davis, CA 95616 USA; 3 Department of Psychiatry, Columbia University, 1051 Riverside Drive, New York, NY 10032 USA

**Keywords:** Sleep duration, Self-reported sleep, Blood pressure, Hypertension, NHANES

## Abstract

**Background:**

The relationship between inadequate sleep duration and hypertension risk has been established in the general population, but there is a gap in the literature on predictors of habitual sleep duration in adults with hypertension. This study examined factors associated with habitual sleep duration among adults with hypertension in the United States (US).

**Methods:**

Data of 5660 adults with hypertension were obtained by combining the 2015–2018 cycles of the National Health and Nutrition Examination Survey (NHANES). Survey weighted multinomial logistic regression models were fit to examine factors associated with short (< 7 h) and long (> 9 h) sleep duration with adequate sleep duration (7–9 h) as the reference.

**Results:**

The prevalence of self-reported adequate sleep duration was 65.7%, while short sleep duration was 23.6%, and long sleep duration 10.7%. Short sleep duration (compared to adequate sleep duration) was positively associated with history of seeking help for sleeping difficulties (relative risk ratio [RRR], 1.25; 95% confidence interval [CI], 1.02–1.53), Non-Hispanic Black race/ethnicity (RRR, 2.08; 95% CI, 1.61–2.67), working ≥45 h/week (RRR, 1.81; 95% CI, 1.32–2.48), and negatively associated with older age ≥ 65 years (RRR, 0.63; 95% CI, 0.45–0.91) and female gender (RRR, 0.70; 95% CI, 0.56–0.88). Long sleep duration was positively associated with female gender (RRR, 1.24; 95% CI, 1.001–1.54), chronic kidney disease (RRR, 1.48; 95% CI, 1.14–1.92), moderate depressive symptoms (RRR, 1.62; 95% CI, 1.08–2.44), moderately severe to severe depressive symptoms (RRR, 1.89; 95% CI, 1.05–3.43), being in retirement (RRR, 3.46; 95% CI, 2.18–5.49), and not working due to health reasons (RRR, 4.87; 95% CI, 2.89–8.22) or other reasons (RRR, 3.29; 95% CI, 1.84–5.88).

**Conclusion:**

This population-based study identified factors independently associated with habitual sleep duration in adults with hypertension. These included help-seeking for sleeping difficulty, gender, age, chronic kidney disease, depressive symptoms, race/ethnicity, and employment status. These findings can help in the development of tailored approaches for promoting adequate sleep duration in adults with hypertension.

**Supplementary Information:**

The online version contains supplementary material available at 10.1186/s12889-021-12465-2.

## Introduction

Sleep is crucial for general wellbeing and physiologic function, including blood pressure (BP) regulation [1]. The American Academy of Sleep Medicine and Sleep Research Society (AASM/SRS) and the National Sleep Foundation (NSF) recommend that individuals aged ≥18 years sleep for a minimum of 7 h a day to stay healthy [1, 2]. However, the proportion of United States (US) adults who habitually sleep less than the recommended hours has increased over the years. For example, the prevalence of habitual short sleep duration (< 7 h/day) among working US adults rose from 30.9% in 2010 to 35.6% in 2018 [3]. The burden of hypertension also remains a public health challenge in the US. Based on the 2017 American College of Cardiology and American Heart Association (ACC/AHA) guidelines, 47.3% of US adults have hypertension, and only 26.1% of those with hypertension have it under control [4].

The 2017 ACC/AHA guidelines recommend weight reduction, adopting a healthy diet, sodium reduction, regular physical activity, and reducing alcohol intake as the primary evidence-based lifestyle interventions for addressing modifiable risk factors of hypertension [5]. However, growing evidence indicates that short sleep duration is also a significant risk factor for hypertension [6–9]. In addition, other studies have reported associations between short sleep duration and factors that can negatively impact hypertension prevention and control, such as obesity [10, 11], unhealthy diet [12, 13], and increased alcohol intake [14]. These findings on the relationship of sleep duration with hypertension and other health-related behaviors indicate the potential role of habitual sleep duration in preventing and controlling hypertension.

Although studies have identified predictors of habitual sleep duration across different US populations [15, 16], none has focused specifically on individuals with hypertension. Given the evidence from many studies of associations between sleep duration and hypertension and its risk factors, it is crucial to examine habitual sleep duration in individuals with hypertension. A better understanding of the determinants of habitual sleep duration in those with hypertension will contribute to more targeted clinical and public health interventions aimed at groups most prone to having inadequate sleep duration. This study’s objective was to identify sociodemographic and health factors independently associated with self-reported habitual sleep duration among US adults with hypertension.

## Methods

### Study population

This cross-sectional study used data collected from US adults aged 18 and above in the 2015–2016 and 2017–2018 waves of the National Health and Nutrition Examination Survey (NHANES). The NHANES is a cross-sectional survey whose aim is to estimate the health and nutritional status of civilian, noninstitutionalized US adults and children of all ages. The survey uses complex stratified multistage sampling to get a nationally representative sample. The data is collected through interviews done in a study participant’s home and physical examinations completed at a designated mobile examination center (MEC) [17].

The 2015–2016 and 2017–2018 NHANES cycles had 19,225 study participants [18]. Of these, there were 11,848 adults aged 18 and older. The primary inclusion criteria for the present study was the presence of hypertension in an adult aged 18 years and above. In the present study, average systolic BP and diastolic BP were calculated from all the recorded BP measurements taken at the MEC. The NHANES participants had three consecutive BP readings taken after 5 min of rest [19]. Based on the 2017 ACC/AHA guidelines [5] and past studies [20, 21], hypertension is defined as the current use of antihypertensive medication in those with a history of hypertension (*n = 3215*), or an average systolic BP ≥130 mmHg (*n = 3744*) or diastolic BP ≥80 mmHg (*n = 2323*). The number of adults who met the criteria of hypertension was 5851.

Out of the remaining 5851, 189 participants were excluded for the following reasons: 7 were pregnant (self-reported pregnant status or a positive urine pregnancy test), 50 were missing data on sleep duration, and 139 only participated in household interviews. Thus, the final study sample of adults with hypertension after exclusions was 5660.

### Outcome measures

The self-reported habitual sleep duration assessment was based on the amount of sleep usually obtained during weekdays or workdays during the main sleep period (day or night). In the 2015–2018 NHANES cycles, the sleep duration was calculated from responses to two survey questions, “What time do you usually fall asleep on weekdays or workdays?” and “What time do you usually wake up on weekdays or workdays?”. In the present study, we categorized habitual sleep duration into short (< 7 h), adequate (7–9 h), and long (> 9 h) based on guidelines by AASM/SRS and NSF [1, 2] and past studies [14, 15].

### Covariate measures

Previous studies [9, 13, 14, 16] and Grandner’s Socio-ecological Model of Sleep and Health [22] were used as a guide in identifying covariates to include in the study. Grandner’s model outlines factors at the “individual, social and societal levels” related to sleep [22]. The current study focused on factors at the individual (e.g., health behaviors and other health-related characteristics) and social level (e.g., socioeconomic status, employment status, and race/ethnicity) of Grandner’s model to identify potential determinants of habitual sleep duration, all measured at the individual level of analysis [22].

The “individual-level” covariates included in this study were gender (male, female), age, body mass index, help-seeking for sleeping difficulty, depressive symptoms, chronic health conditions, cigarette smoking, alcohol intake, and physical activity. Age was categorized as 18–44 years (young adults), 45–64 years (middle-aged adults), and ≥ 65 years (older adults). Body mass index (BMI) was categorized as < 25 (no overweight or obesity), 25 to < 30 (overweight), 30 to < 35 (obesity class I), 35 to < 40 (obesity class II), and ≥ 40 kg/m^2^ (obesity class III), based on the World Health Organization (WHO) guidelines [23]. Help-seeking for sleeping difficulty was categorized as yes or no based on the response to the question “Have you ever told a doctor or other health professional that you have trouble sleeping?” Depressive symptoms were screened using the 9-item Patient Health Questionnaire (PHQ-9, possible range of 0–27), categorized based on the established criteria of minimal or none (0–4), mild (5–9), moderate (10–14), and moderately severe to severe (≥15) depressive symptoms [24].

Chronic health conditions were assessed based on either self-reported history (being informed by a doctor or other health professional of having the specific health condition) or laboratory test findings done at the mobile examination centers. Stroke, heart disease (coronary heart disease, angina, heart attack, or congestive heart failure), arthritis (including gout), and chronic obstructive pulmonary disease (including chronic bronchitis and emphysema) were self-reported. Those who answered in the affirmative to both “Has a doctor or health professional ever told you that you have asthma?” and “Do you still have asthma?” were classified as having asthma. Diabetes was defined as either a self-reported history of diabetes or blood glycohemoglobin ≥6.5%. Chronic kidney disease (CKD) was defined as self-reported history of undergoing dialysis in the previous 12 months or an estimated glomerular filtration rate (eGFR) less than 60 mL/min/1.73 m^2^ computed using the Chronic Kidney Disease Epidemiology Collaboration (CKD-EPI) equation [25, 26].

Smoking status was categorized as never smoker (never smoked at least 100 cigarettes in their lifetime), former smoker (smoked at least 100 cigarettes in their lifetime but not smoking currently), and current smoker (smokes cigarettes some days or every day). Alcohol intake was based on the self-reported average number of alcoholic drinks in a lifetime and the past year. The equivalent for one alcohol drink used in NHANES was a 12-oz beer, a 5-oz glass of wine, or one-and-half ounces of liquor. Based on the 2015–2020 US dietary guidelines, alcohol intake was categorized as none (never had at least 12 alcoholic drinks in a lifetime or any alcohol in the past year), moderate (one drink for women and not more than two drinks for men in a day), and heavy (more than one or two drinks in a day for women and men, respectively) [27].

Physical activity levels were assessed by calculating the metabolic equivalent of task (MET) minutes per week from the self-reported minutes of moderate-to-vigorous intensity physical activity related to leisure-time, work, and transportation (commuting by walking or bicycling). MET scores are used to estimate an activity’s intensity, and one MET is defined as the energy expenditure at rest [28]. The MET scores proposed by NHANES for moderate-intensity, vigorous-intensity, and transportation-related physical activity are four, eight, and four, respectively [29]. MET-minutes per week were computed by multiplying the minutes per week of each of the three categories of physical activity (moderate-intensity, vigorous-intensity, and transportation-related physical activity) with their respective MET scores. The MET minutes for the three activities were then summed to compute the total MET minutes per week. The US guidelines on physical activity recommend that adults get at least 75 to 150 min of vigorous-intensity or 150 to 300 min of moderate-intensity physical activity weekly [30]. The physical activity level was categorized as none, low, sufficient, and high to correspond to zero, < 600, 600–1200, and > 1200 MET-minutes a week, respectively [28].

The “social-level” covariates were all measured at the individual level of analysis and included race/ethnicity (non-Hispanic White, non-Hispanic Black, Hispanic, non-Hispanic Asian, Other), nativity status (US-born, not US-born), marital status (married or living with a partner, unmarried [i.e., never married, separated, divorced, or widowed]), education level (less than high school, high school graduate, some college, college graduate), health insurance (insured, uninsured), employment status, and family income to poverty ratio. Employment status was assessed based on responses to the following survey items: (1) the type of work done the previous week; (2) the number of hours worked in all jobs or businesses the previous week; (3) whether one usually worked 35 h per week; and (4) the main reason one did not work the previous week. The categories of employment status were: works < 35 h/week, works 35–44 h/week, works ≥45 h/week, not working because they are retired, not working due to health reasons, and not working due to other reasons [for example, taking care of family, attending school, or laid off from work]). Family income to poverty ratio was categorized as < 1.00, 1.00–1.99, 2.00–3.99, and ≥ 4.00.

### Statistical analysis and missing data

In the final analytic sample (*n = 5660*), 20.1% of the observations had missing data in one or more covariates. The degree of missingness (unweighted number and weighted percentages) was as follows: marital status (*n = 39, 0.4%*), education level (*n = 9, 0.1%),* health insurance status *(n = 10, 0.2%),* cigarette smoking status *(n = 6, 0.1%),* alcohol intake *(n = 403, 5.7%),* BMI *(n = 90, 1.3%),* depressive symptoms *(n = 357, 5.2%),* and income to poverty ratio *(n = 689, 9.8%)*. Imputation of the missing data was completed using multiple imputation by chained equations (MICE) [31].

All variables in the study (covariates and outcome variable) and NHANES design variables (strata, cluster, and survey weights) were included in the imputation model to avoid generating biased estimates in the analysis model [30, 32]. The literature on multiple imputation also recommends having in the imputation model other variables associated with missingness of data to make multiple imputations under the assumptions of missing at random assumptions more plausible [30, 33]. The auxiliary variables included in the imputation model included the reference person’s age and education level, household size, number of rooms in the household, and homeownership. Stata’s *mi impute chained* command was used to implement the multiple imputation. A total of 40 imputed datasets were generated, and Stata’s *mi estimate* command was used to analyze and pool the results [34].

We did a sensitivity analysis to check for differences between the observed (pre-imputation) data and the complete imputed data (post-imputation) by comparing the subgroup proportions across the variables with missing data. There was minimal or no change in the distribution of pre-imputed and post-imputed data across marital status, education level, health insurance status, BMI, smoking status, depressive symptoms, and alcohol intake. Changes were noted in the income to poverty ratio variable. The proportion of those in the ≥4.00 category decreased from 36.6 to 35.6% after imputation. The proportion of adults in the < 1.00 group increased from 12.5 to 13.1%, and the 1.00–1.99 group increased from 21.1 to 21.7% (Additional file 1: Supplemental Table 1). The results presented in this report are based on analyses of the complete imputed data.

Survey analysis procedures incorporating the NHANES sample’s MEC examination weights and strata and cluster variables were used to account for NHANES’s sampling procedures and non-response to obtain results representative of the sampled population [17]. Descriptive analysis was conducted on all covariates for the total study sample. In the bivariate analyses, the distribution of all covariates across the categories of habitual sleep duration was assessed using the χ^2^ test.

Since the outcome variable (habitual sleep duration) categories are ordered, we examined the ordinal logistic regression model for model fit and violation of statistical assumptions*.* The approximate likelihood-ratio test of proportionality of odds across response categories and parallel regression assumption test statistics were significant indicating a violation of proportionality of odds assumption. Consequently, a multinomial logistic regression model was used in the analyses. We fit the model using the complete imputed data with habitual sleep duration as the outcome of interest. The multinomial logistic regression models (unadjusted and adjusted) were fitted with all covariates to examine their associations with short (< 7 h) and long sleep duration (> 9 h) using adequate sleep duration (7–9 h) as the reference category. The multinomial logistic regression model results were compared with complete case analysis results. All data were analyzed using STATA 1C Version 15 [35]. The level of significance for all analyses was set at a *p*-value < 0.05.

## Results

### Sample population characteristics

The average age of the participants was 56.1 years; 52.1% were male; 64.1% were non-Hispanic White, 13.3% non-Hispanic Black and 12.9% Hispanic; 33.2% had some college education, and 26.9% were college graduates. The prevalence of self-reported adequate sleep duration was 65.7%, while short sleep duration was 23.6%, and long sleep duration was 10.7%. Over a third (37.2%) had a history of ever telling a doctor or other health professional that they had trouble sleeping. Most (89.8%) had health insurance. Over half (51.3%) had a BMI of 30 kg/m^2^ and above; 48.3% had high self-reported physical activity levels, while 26.2% were physically inactive. The prevalence of heavy alcohol intake was 32.0% (Tables 1 and 2).

**Table 1:** Individual-level characteristics by sleep duration from NHANES (2015–2018) sample of US adults with hypertension ^a^

Characteristics		Sleep duration			
	Overall	Short	Adequate	Long	***P***-value
**Total, n (%)**	5660 (100%)	1444 (23.6)	3495 (65.7)	721 (10.7)	
**Sex, %**					
Male,	2927 (52.1)	60.0	51.3	39.7	< 0.001
Female	2733 (47.9)	40.0	48.7	60.3	
**Age, mean years (SE)**	56.1 (0.4)	53.7 (0.5)	56.2 (0.5)	60.4 (0.8)	
**Age category, %**					
18–44	1159 (23.8)	26.3	23.6	18.9	< 0.001
45–64	2356 (43.7)	51.8	42.5	33.3	
65 and above	2145 (32.5)	21.9	33.9	47.8	
**Comorbidities, %**					
Heart disease	763 (11.4)	12.3	10.4	15.5	0.073
Stroke	361 (5.1)	4.5	4.6	9.6	< 0.001
COPD or asthma	847 (14.4)	15.3	13.1	20.1	0.006
Arthritis	2365 (41.8)	40.6	40.7	50.9	0.005
Diabetes mellitus	1547 (22.1)	22.8	20.6	30.1	0.015
Chronic kidney disease	745 (11.2)	9.5	10.4	20.1	< 0.001
**Depressive symptoms, %**					
Minimal or none	3933 (74.7)	71.7	77.7	62.4	< 0.001
Mild	890 (16.5)	16.9	15.4	22.6	
Moderate	296 (5.8)	7.5	4.7	8.7	
Moderately severe to severe	184 (3.0)	3.9	2.2	6.3	
**Help-seeking for sleeping difficulty, %**	1859 (37.2)	41.0	35.5	39.8	0.066
**BMI category, %**					
< 25	1109 (18.1)	17.2	18.1	20.2	0.439
25 - < 30	1755 (30.5)	29.2	31.0	30.7	
30 - < 35	1349 (25.7)	25.0	25.9	26.6	
35 - < 40	737 (13.8)	14.5	14.1	10.9	
≥ 40	620 (11.8)	14.1	10.9	11.7	
**Smoking status, %**					
Never smoker	3066 (52.8)	50.6	54.2	48.9	< 0.001
Current smoker	1005 (17.2)	22.1	14.7	21.3	
Former smoker	1583 (30.0)	27.3	31.1	29.8	
**Alcohol intake, %**					
None	1973 (29.4)	29.8	27.6	39.2	0.001
Moderate	1827 (38.6)	35.8	40.4	34.4	
Heavy	1457 (32.0)	34.4	32.0	26.4	
**Physical activity level, %**					
Sufficient	642 (11.7)	10.3	12.4	10.6	< 0.001
None	1770 (26.2)	26.0	24.3	38.6	
Low	803 (13.7)	10.6	15.0	12.8	
High	2445 (48.3)	53.1	48.3	38.0	

Abbreviations: *SE* standard error, *COPD* chronic obstructive pulmonary disease, *BMI* body mass index

^a^Data are expressed as an unweighted number of participants and weighted percentages and means

**Table 2:** Social-level characteristics by sleep duration from NHANES (2015–2018) sample of US adults with hypertension^a^

Characteristics		Sleep duration			
	Overall	Short	Adequate	Long	***P***-value
**Total, n (%)**	5660 (100%)	1444 (23.6)	3495 (65.7)	721 (10.7)	
**Race/Ethnicity, %**					
Non-Hispanic White	1905 (64.1)	56.4	66.9	63.6	< 0.001
Non-Hispanic Black	1474 (13.3)	19.4	10.9	14.4	
Hispanic	1381 (12.9)	13.9	12.4	13.8	
Non-Hispanic Asian	652 (5.4)	5.3	5.6	4.7	
Other	248 (4.3)	5.0	4.2	3.5	
**Marital status, %**					
Married/Living with partner	3301 (63.1)	61.7	65.1	54.3	0.005
Unmarried	2320 (36.9)	38.3	34.9	45.7	
**Nativity status, %**					
US-born	3964 (83.2)	81.6	83.3	85.4	0.211
Not US-born	1696 (16.8)	18.4	16.7	14.6	
**Education Level, %**					
College graduate	1215 (26.9)	23.4	29.6	18.3	< 0.001
Some college	1732 (33.2)	36.2	33.5	24.6	
High school graduate	1354 (26.0)	26.1	24.7	33.5	
Less than high school	1350 (13.9)	14.3	12.2	23.6	
**Income to poverty ratio, %**					
≥ 4.00	1165 (36.6)	32.9	40.2	22.3	< 0.001
2.00–3.99	1373 (29.8)	33.7	29.0	26.5	
1.00–1.99	1447 (21.1)	20.2	20.2	28.8	
< 1.00	986 (12.5)	13.2	10.6	22.4	
**Employment Status, %**					
Works 35–44 h/week	1184 (23.8)	23.3	26.2	10.3	< 0.001
Works < 35 h/week	670 (12.1)	9.9	13.3	9.2	
Works ≥45 h/week	815 (18.5)	28.3	17.4	3.9	
Not working – health reasons	743 (10.3)	11.1	8.1	22.4	
Not working - retired	1623 (26.5)	20.5	26.5	39.7	
Not working – other reasons	625 (8.8)	6.9	8.5	14.5	
**Has health insurance, %**					
Yes	4965 (89.8)	86.3	91.1	89.6	0.003
No	685 (10.2)	13.7	8.9	10.4	

^a^Data are expressed as an unweighted number of participants and weighted percentages

In bivariate descriptive comparisons, participants with short sleep duration were younger and more likely to be male, non-Hispanic Black, have no health insurance, and work ≥45 h weekly. Conversely, individuals with long sleep duration were older and more likely to be female, not employed, have a high school or lower level of education, have comorbidity or moderate to severe depressive symptoms, and be non-drinkers and physically inactive (Tables 1 and 2).

### Individual-level and social-level factors associated with habitual sleep duration

The multinomial logistic analysis results shown in Tables 3 were derived from the complete data (*n* = 5660) created using multiple imputations, and all associations are presented relative to adequate sleep duration. In the unadjusted multinomial logistic regression results, all covariates except BMI and nativity status showed significant associations with habitual sleep duration (Table 3).

**Table 3:** Multinomial logistic regression - factors associated with habitual sleep duration in US adults with hypertension

	Short (vs. adequate) sleep duration				Long (vs. adequate) sleep duration			
	Crude RRR (95%CI) ^**a**^	***P***	RRR (95% CI) ^**b**^	***P***	Crude RRR (95%CI) ^**a**^	***P***	RRR (95% CI) ^**b**^	***P***
***Individual-level factors***								
**Age**								
18–44	REF		REF		REF		REF	
45–64	1.09 (0.86–1.39)	0.45	1.20 (0.96, 1.51)	0.11	0.98 (0.68–1.41)	0.90	0.72 (0.46–1.13)	0.15
≥ 65	0.58 (0.47–0.72)	< 0.001	**0.63 (0.45–0.91)**	**0.01**	1.76 (1.31–2.36)	< 0.001	0.74 (0.50–1.09)	0.12
**Female (vs. Male)**	0.70 (0.58–0.85)	0.001	**0.70 (0.56, 0.88)**	**0.003**	1.60 (1.26–2.03)	< 0.001	**1.24 (1.00–1.54)**	**0.049**
**Comorbidities**								
Heart disease (vs. no heart disease)	1.20 (0.82–1.76)	0.33	1.20 (0.80–1.78)	0.36	1.57 (1.15–2.17)	0.01	0.88 (0.59–1.30)	0.50
Stroke (vs. no stroke)	0.97 (0.64–1.48)	0.89	0.79 (0.54–1.16)	0.21	2.18 (1.56–3.04)	< 0.001	1.09 (0.78–1.54)	0.60
COPD or asthma (vs. no COPD or asthma)	1.19 (0.90–1.58)	0.20	1.10 (0.83–1.47)	0.49	1.67 (1.23–2.26)	0.002	0.98 (0.72–1.35)	0.91
Arthritis (vs. no arthritis)	0.99 (0.83–1.19)	0.96	1.06 (0.89–1.26)	0.52	1.51 (1.17–1.95)	0.002	0.93 (0.71–1.21)	0.60
Diabetes mellitus (vs. no diabetes mellitus)	1.14 (0.84–1.52)	0.36	1.08 (0.83–1.40)	0.55	1.66 (1.22–2.25)	0.002	1.37 (1.00–1.88)	0.05
CKD (vs. no CKD)	0.90 (0.68–1.21)	0.49	1.18 (0.86–1.61)	0.31	2.18 (1.70–2.79)	< 0.001	**1.48 (1.14–1.92)**	**0.01**
**Depressive symptoms**								
Minimal or none	REF		REF		REF		REF	
Mild	1.21 (0.94–1.56)	0.13	1.12 (0.88–1.44)	0.34	1.83 (1.32–2.53)	0.001	1.35 (0.95–1.93)	0.09
Moderate	1.73 (1.08–2.77)	0.03	1.61 (0.99–2.61)	0.05	2.32 (1.63–3.30)	< 0.001	**1.62 (1.08–2.44)**	**0.02**
Moderately severe to severe	1.91 (1.13–3.23)	0.02	1.66 (0.88–3.14)	0.11	3.62 (2.07–6.34)	< 0.001	**1.89 (1.05–3.43)**	**0.04**
**Help-seeking for sleeping difficulty**	1.26 (1.02–1.56)	0.03	**1.25 (1.02–1.53)**	**0.03**	1.20 (0.91–1.59)	0.18	0.84 (0.61–1.16)	0.28
**Body mass index (BMI)**								
< 25	REF		REF		REF		REF	
25 - < 30	0.99 (0.71–1.38)	0.94	0.98 (0.70–1.37)	0.88	0.89 (0.65–1.21)	0.44	1.00 (0.72–1.39)	0.99
30 - < 35	1.01 (0.70–1.46)	0.95	0.93 (0.65–1.34)	0.70	0.92 (0.61–1.39)	0.68	1.06 (0.69–1.62)	0.78
35 - < 40	1.08 (0.75–1.55)	0.67	1.02 (0.70–1.48)	0.93	0.71 (0.44–1.12)	0.14	0.71 (0.42–1.22)	0.21
≥ 40	1.34 (0.99–1.83)	0.06	1.19 (0.85–1.66)	0.30	0.96 (0.63–1.46)	0.83	0.91 (0.56–1.45)	0.67
**Alcohol intake**								
None	REF		REF		REF		REF	
Moderate	0.83 (0.66–1.05)	0.11	0.84 (0.67–1.05)	0.11	0.60 (0.47–0.78)	< 0.001	0.99 (0.76–1.30)	0.96
Heavy	1.00 (0.77–1.30)	0.99	0.89 (0.68–1.17)	0.38	0.58 (0.43–0.78)	0.001	0.91 (0.64–1.31)	0.61
**Cigarette smoking**								
Never smoker	REF		REF		REF		REF	
Current smoker	1.61 (1.24–2.08)	0.001	1.25 (0.93–1.68)	0.14	1.60 (1.13–2.29)	0.01	1.16 (0.82–1.63)	0.39
Former smoker	0.94 (0.76–1.16)	0.55	0.88 (0.72–1.07)	0.19	1.06 (0.79–1.42)	0.67	0.96 (0.72–1.29)	0.78
**Physical activity level**								
Sufficient	REF		REF		REF		REF	
None	1.28 (0.96–1.70)	0.09	1.18 (0.89–1.57)	0.23	1.85 (1.27–2.70)	0.002	1.39 (0.90–2.13)	0.13
Low	0.85 (0.60–1.20)	0.33	0.82 (0.59–1.15)	0.25	1.00 (0.66–1.50)	0.99	0.91 (0.59–1.40)	0.65
High	1.32 (0.98–1.77)	0.07	1.14 (0.85–1.54)	0.37	0.92 (0.57–1.46)	0.71	1.05 (0.65–1.71)	0.84
***Social-level factors***								
**Race/Ethnicity**								
Non-Hispanic White	REF		REF		REF		REF	
Non-Hispanic Black	2.11 (1.70–2.61)	< 0.001	**2.05 (1.59–2.63)**	**< 0.001**	1.38 (0.99–1.92)	0.05	1.13 (0.81–1.57)	0.47
Hispanic	1.33 (1.06–1.67)	0.02	1.16 (0.82–1.64)	0.39	1.17 (0.89–1.52)	0.24	1.08 (0.80–1.46)	0.62
Non-Hispanic Asian	1.13 (0.87–1.46)	0.34	1.16 (0.74–1.81)	0.51	0.89 (0.65–1.21)	0.44	1.12 (0.68–1.84)	0.65
Other	1.42 (0.97–2.09)	0.07	1.24 (0.79–1.95)	0.34	0.89 (0.51–1.56)	0.68	0.78 (0.48–1.28)	0.31
**Marital status**								
Married/Living with partner	REF		REF		REF		REF	
Unmarried	1.16 (0.95–1.42)	0.15	1.14 (0.95–1.37)	0.14	1.56 (1.20–2.04)	0.002	1.06 (0.78–1.45)	0.71
**Nativity**								
US-born	REF		REF		REF		REF	
Not US-born	1.12 (0.90–1.40)	0.28	1.16 (0.83–1.63)	0.37	0.86 (0.65–1.12)	0.24	0.71 (0.47–1.07)	0.10
**Education Level**								
College graduate	REF		REF		REF		REF	
Some college	1.36 (1.01–1.84)	0.04	1.44 (0.92–1.68)	0.15	1.19 (0.69–2.05)	0.34	0.85 (0.51–1.43)	0.53
High school graduate	1.33 (0.98–1.81)	0.07	1.17 (0.85–1.60)	0.32	2.20 (1.24–3.88)	0.01	1.42 (0.86–2.34)	0.17
Less than high school	1.47 (1.10–1.97)	0.01	1.21 (0.88–1.67)	0.24	3.14 (1.76–5.63)	< 0.001	1.59 (0.88–2.89)	0.12
**Income to poverty ratio**								
≥ 4.00	REF		REF		REF		REF	
2.00–3.99	1.38 (1.07–1.79)	0.02	1.21 (0.95–1.56)	0.12	1.64 (0.99–2.73)	0.06	1.20 (0.74–1.95)	0.45
1.00–1.99	1.20 (0.92–1.55)	0.17	0.94 (0.69–1.28)	0.70	2.53 (1.52–4.19)	0.001	1.28 (0.77–2.14)	0.33
< 1.00	1.47 (1.09–1.97)	0.01	0.97 (0.67–1.39)	0.85	3.70 (2.11–6.46)	< 0.001	1.45 (0.82–2.58)	0.19
**Has health insurance**								
Yes	REF		REF		REF		REF	
No	1.64 (1.24–2.17)	0.001	1.30 (0.97–1.75)	0.08	1.19 (0.80–1.76)	0.37	1.09 (0.75–1.59)	0.65
**Employment Status**								
Works 35–44 h/week	REF		REF		REF		REF	
Works < 35 h/week	0.84 (0.58–1.20)	0.32	0.88 (0.61–1.28)	0.50	1.77 (1.15–2.73)	0.01	**1.71 (1.07–2.73)**	**0.03**
Works ≥45 h/week	1.83 (1.34–2.51)	0.001	**1.81 (1.32–2.48)**	**0.001**	0.58 (0.28–1.19)	0.13	0.66 (0.34–1.29)	0.21
Not working – Health reasons	1.55 (1.10–2.18)	0.02	1.12 (0.77–1.63)	0.53	7.09 (4.62–10.9)	< 0.001	**4.87 (2.89–8.22)**	**< 0.001**
Not working - Retired	0.87 (0.69–1.09)	0.21	**1.41 (1.01–1.98)**	**0.04**	3.82 (2.42–6.04)	< 0.001	**3.46 (2.18–5.49)**	**< 0.001**
Not working - Other reason	0.91 (0.70–1.19)	0.48	0.85 (0.65–1.12)	0.23	4.33 (2.66–7.05)	< 0.001	**3.29 (1.84–5.88)**	**< 0.001**

Abbreviations: *RRR* relative risk ratio, *P p*-value, *COPD* chronic obstructive pulmonary disease

^a^Unadjusted model

^b^Adjusted model – each covariate adjusted for all other variables in the table

In the fully adjusted multinomial logistic model, age, sex, history of help-seeking for sleeping difficulty, depressive symptoms, and CKD were associated with habitual sleep duration. Older adults (≥ 65 years old) were less likely than young adults (18–44 years) to have short sleep duration (RRR, 0.63; 95% CI, 0.45–0.91). No age differences were noted in odds for being a long sleeper. Compared to men, women were less likely to be short sleepers (RRR, 0.70; 95% CI, 0.56–0.88) and more likely to be long sleepers (RRR, 1.24; 95% CI, 1.001–1.54). Those reporting a history of ever telling a doctor or other healthcare professional they had trouble sleeping were more likely to have short sleep duration (RRR, 1.25; 95% CI, 1.02–1.53).

Long sleep duration was significantly associated with moderate depressive symptoms (RRR, 1.62; 95% CI, 1.08–2.44) and moderately severe to severe depressive symptoms (RRR, 1.89; 95% CI, 1.05–3.43) compared to minimal or no depressive symptoms. In addition, those with CKD were more likely to have a long sleep duration than those with no CKD (RRR, 1.48; 95% CI, 1.14–1.92). Other comorbid conditions (diabetes mellitus, COPD or current asthma, heart disease, stroke, and arthritis) were not independently associated with habitual sleep duration (Table 3). Similarly, cigarette smoking status, alcohol intake, BMI, and total physical activity level were not independently associated with habitual sleep duration. The physical activity and sleep duration relationship findings remained the same when total physical activity level was substituted with physical activity derived from leisure-time and transport-related activities in the multinomial logistic model.

Non-Hispanic Black adults with hypertension were twice as likely as their non-Hispanic White counterparts to have short sleep duration (RRR, 2.08; 95% CI, 1.61–2.67). The odds of being a short or long sleeper in Hispanic, non-Hispanic Asian, and Other race/ethnic groups were not significantly different from that of the non-Hispanic White race/ethnic group (Table 3).

In the association between employment status and habitual sleep duration, individuals working for ≥45 h/week were more likely to have short sleep duration than those working 35–44 h/week (RRR, 1.81; 95% CI, 1.32–2.48). Those retired were more likely to be short sleepers (RRR, 1.41; 95% CI, 1.01–1.98) or long sleepers (RRR, 3.46; 95% CI, 2.18–5.49) than those working 35–44 h/week. Long sleep duration was also more likely in those not working due to health reasons (RRR, 4.87; 95% CI, 2.89–8.22) and those not working due to other reasons such as attending school and taking care of the family (RRR, 3.29; 95% CI, 1.84–5.88) compared to those working 35–44 h/week. Marital status, education level, and health insurance did not show independent associations with habitual sleep duration.

The adjusted multinomial logistic regression results from the complete imputed data model (*n = 5660*) were compared to those of complete case analysis (*n = 4520*). The direction and significance of the relationship between the covariates and habitual sleep duration in the two models were similar to except in employment status, help-seeking for sleeping difficulty, and depressive symptoms covariates where the level of significance differed (Additional file 2: Supplemental Table 2). In employment status, retirement was associated with short sleep in the imputed model (RRR, 1.41; 95% CI, 1.01–1.98) but not in complete case analysis (RRR, 1.39; 95% CI, 0.94–2.06). Help-seeking for sleeping difficulty was associated with short sleep in the imputed model (RRR,1.25; 95% CI, 1.02–1.53) but not in complete case analysis (RRR,1.20; 95% CI, 0.97–1.50). Finally, moderate depressive symptoms were associated with short sleep duration in the complete case analysis (RRR, 1.87; 95% CI, 1.23–2.85) but not in the imputed model (RRR, 1.61; 95% CI, 0.99–2.61).

Results from the complete case analyses showed no significant associations between depressive symptoms and long sleep duration, but in the complete imputed data model, long sleep duration was associated with both moderate (RRR,1.62; 95% CI, 1.08–2.44 versus RRR, 1.52; 95% CI, 0.98–2.37 in the complete case analysis) and moderately severe to severe depressive symptoms (RRR,1.89; 95% CI, 1.05–3.43 vs. RRR, 1.78; 95% CI, 0.84–3.76 in the complete case analysis). The results show that the differences noted were on the significance of the relationship between the specified variables and habitual sleep duration but not on the direction of the relationships. The complete case analysis model included 4520 observations (compared to 5660 observations for complete imputed data), translating to a 20% loss in information. This loss of information may bias results in the complete case analysis and may have contributed to the differences noted between the two models. Some of the differences observed could also be due to an increase in power in the imputed data model.

## Discussion

In this large nationally representative sample of adults with hypertension, we find numerous sociodemographic and health characteristics are significantly associated with habitual sleep duration. Among US adults with hypertension, help-seeking for sleeping difficulty, defined in the present study as a history of telling a doctor or other health professional that one had trouble sleeping, was independently associated with short sleep duration. Previous research has also noted significant associations between self-reported difficulties initiating or maintaining sleep and short sleep duration [36]. These findings have important implications because short sleep duration and other sleep problems, such as insomnia synergistically increase the risk for adverse cardiovascular outcomes [37, 38]. The significant association between help-seeking for sleeping difficulty and short sleep duration among those with hypertension provides a point of intervention for health care teams to comprehensively assess and support all aspects of sleep health, including habitual sleep duration, in patients who report sleep problems.

Findings from studies with other populations showed no differences by gender in habitual sleep duration [13, 14]. In contrast, we found that women with hypertension were less likely to be short sleepers and more likely to be long sleepers than men with hypertension. The inconsistency may be partly due to the different approaches used in measuring sleep duration. The two studies defined sleep duration based on the amount of sleep obtained in a day [14] or 24 h [13], increasing the likelihood that napping time was captured. In contrast, our study measured the amount of sleep obtained in the main sleep period. Studies have reported a higher frequency of napping among men [39, 40]. The differences in napping may account for sex differences in sleep duration when the assessment is limited to the amount of sleep obtained during the main sleep period.

The relationship between chronic disease and sleep duration varies across studies. Consistent with other studies [41, 42], we found that CKD was associated with long sleep duration but not short sleep duration. Other previous research findings found that both short (< 6 h/night) and long sleep duration (> 8 h/night) were positively associated with incident CKD with the strongest association occurring in those who habitually slept < 4 h per night [43]. The increased risk of CKD in short sleepers may be due to the increased sympathetic nervous system activity and inflammation that occurs with sleep loss [44].

On the other hand, there is minimal evidence of a pathway through which long sleep duration can cause disease. In those with chronic disease, the long sleep duration may be due to ill health or the underlying pathophysiological mechanisms of the specific condition, such as increased inflammation [1, 45]. The inflammatory processes can cause excessive fatigue and sleepiness [45, 46] and contribute to more time spent in bed. Therefore, the inclusion of markers of pathophysiological processes such as inflammatory markers in future studies would provide more insight into the mechanisms underlying the relationship between disease and sleep duration.

Similar to our findings in adults with hypertension, many previous studies in the general population have found no association between habitual sleep duration and COPD, heart disease, diabetes [16, 47], stroke [16, 47], or arthritis [13, 16]. However, other studies found that short sleep duration was significantly associated with COPD [13], heart disease [13, 14], and arthritis [47], while stroke was associated with long sleep duration [43]. The inconsistencies across studies may be partly related to differences in baseline characteristics of study participants, the varied ways in which sleep duration is measured and analyzed, differences in the proportion of those with comorbidities, and other factors controlled in the studies.

Consistent with prior findings across different adult populations, including older adults and those attending outpatient care settings [16, 36, 48, 49], we found that moderate to severe depressive symptoms were associated with long sleep duration. Other findings have also shown an association between sleeping less than 6 h and risk for depression [49] which may point to a dose-response relationship between short sleep and depression. Overall, the literature points to a bidirectional relationship between sleep duration and mental health indicators, including depressive symptoms. Circadian rhythm disruptions and insomnia are common in depression and can contribute to short sleep [50]. Sleep loss has also been linked to impairment in emotional regulation and decreased positive and increased negative emotions, which can trigger and worsen depressive symptoms [51, 52]. Depression has also been linked to elevated levels of pro-inflammatory markers [53, 54], whose effects include excessive fatigue and sleepiness [46, 54, 55]. Hypertension is also associated with increased inflammation [56, 57]. Therefore, having both hypertension and depression may worsen the effects of inflammation in the body.

The relationship between sleep duration and other health-related behaviors has been examined in many studies. Consistent with prior studies, we found no significant relationship between alcohol intake and habitual sleep duration [13, 16]. Other previous findings have shown heavy alcohol intake to be associated with sleep continuity disturbances but not sleep duration [58]. Alcohol intake near bedtime can affect sleep quality because it disrupts the normal sleep architecture [59]. Consistent with previous studies, we found no association between cigarette smoking and habitual sleep duration [13, 16].

Research findings on the relationship between physical activity and sleep duration are mixed. Similar to a prior study in older adults [13], we noted no associations between physical activity and sleep duration in adults with hypertension. Other findings suggest that being physically active is associated with reduced odds of having a long sleep duration [14, 60]. The negative association between physical activity and long sleep duration may be related to other confounding factors, such as health conditions, not controlled for in studies. Findings from experimental studies suggest that physical activity substantially improves other sleep parameters such as sleep regularity and sleep quality but only has modest effects on total sleep time [61, 62]. On the other hand, adequate sleep can enhance the ability to remain physically active because it improves mental and physical performance [63].

We noted that individuals working ≥45 h per week were more likely to be short sleepers, while those working < 35 h per week or not working were more likely to be long sleepers when compared to individuals working 35–44 h per week. Previous studies also have found significant associations between longer work hours and short sleep duration [64, 65]. Findings from the American Time Use Survey showed that the main activity exchanged for sleep is paid work [66], and other results show that a reduction in work hours leads to a significant increase in sleep duration [65]. In the present study, those in retirement also had a slight but significant increase in odds of being a short sleeper than those working 35–44 h/week. These findings on retirement and sleep duration may be partly explained by how sleep duration was quantified in our study. The present study only considered sleep obtained during the main sleep period. Studies have found that individuals who have transitioned from work to retirement tend to nap more frequently or for longer durations when compared to those who are employed [67, 68].

Our results on the relationship between race/ethnicity and habitual sleep duration among adults with hypertension are partly consistent with previous findings in the general US adult population. Consistent with other studies [69–71], we found that non-Hispanic Black adults were more likely than non-Hispanic White adults to be short sleepers. However, prior studies also reported significantly higher odds for short sleep duration among Hispanic [71] and non-Hispanic Asian adults than non-Hispanic White adults [70, 71]. In contrast, our study showed that the odds of being a short or long sleeper in Hispanic or non-Hispanic Asian adults were not significantly different from that of non-Hispanic White adults. Of note, in these studies, the strongest association between race/ethnicity and short sleep duration has been observed in non-Hispanic Black adults [70, 71].

Previous studies have also found objectively measured total sleep time of non-Hispanic Black US adults to be significantly shorter than that of non-Hispanic White, Hispanic, and non-Hispanic Asian US adults [69, 71]. These findings on non-Hispanic Black race/ethnicity and short sleep duration are noteworthy because hypertension is more prevalent among non-Hispanic Black adults than in non-Hispanic White, Hispanic, or non-Hispanic Asian adults [20]. Previous findings suggest that the racial/ethnic differences in sleep duration may be related to differences in stress and perceived racial discrimination [15, 72]. Further research is needed to explore further the mechanisms underlying these racial/ethnic differences in sleep duration.

Despite this study being nationally representative, there are limitations to be noted. The study was cross-sectional, and consequently, we cannot infer any temporality of the relationships between predictors such as chronic health conditions and habitual sleep duration. Second, NHANES did not have data on other potential predictors of sleep duration, such as inflammatory biomarkers, stress, sleep attitudes, napping, and caregiving [15, 16, 39, 73, 74]. Third, data for many variables used in this study, including habitual sleep duration, chronic health conditions, and health behaviors, were measured using self-reports. Even though the collection of self-reported data is more feasible than objective measurements in large-scale surveys such as NHANES, it increases the risk for measurement error related to factors such as poor recall and social desirability [75]. To reduce measurement error, part of the NHANES protocols involved clarifying with participants any extreme high or low self-reports on various questionnaire items. For example, for sleep duration, NHANES interviewers verified with participants sleep duration ≤4 or ≥ 10 h.

Despite the limitations noted, the information gathered on factors associated with short and long sleep duration in adults with hypertension provides important contributions to the literature on predictors of poor sleep outcomes in this population. Future studies in hypertensive adults should consider using objective measures of sleep duration and other health behaviors, such as physical activity, and include other important predictors of sleep duration in their analyses.

## Conclusion

This study used data from a national survey to assess the contribution of sociodemographic and health factors related to habitual sleep duration in adults with hypertension. In adults with hypertension, the factors positively associated with short sleep duration included help-seeking for sleeping difficulty, non-Hispanic Black race/ethnicity, working 45 or more hours per week, and being in retirement. These factors provide information about hypertensive adults at most risk for poor health outcomes related to inadequate sleep’s adverse effects. Such evidence can help develop tailored approaches for supporting healthy sleep habits, including adequate habitual duration, in adults with hypertension. In addition, future intervention studies aimed at promoting healthy sleep habits in groups with high rates of short sleep duration will help identify how sleep impacts health outcomes. Also, intervention studies in adults with hypertension are needed to identify modifiable factors that improve habitual sleep duration.

## Supplementary Information

**Additional file 1: Supplemental Table 1.** Comparative sensitivity analysis between pre-imputation and post-imputation data of variables with missing data.

**Additional file 2: Supplemental Table 2.** Comparison of results on factors associated with short and long sleep duration – complete case analysis and post-imputation.

## Data Availability

The datasets generated and/or analyzed during the current study are available in the Centers for Disease Control and Prevention (CDC) NHANES repository, https://wwwn.cdc.gov/nchs/nhanes/.

## Abbreviations

AASM/SRS: American Academy of Sleep Medicine and Sleep Research Society
ACC/AHA: American College of Cardiology and American Heart Association
BP: Blood pressure
BMI: Body mass index
CDC: Centers for Disease Control and Prevention
COPD: Chronic obstructive pulmonary disease
CI: Confidence interval
CKD: Chronic kidney disease
eGFR: Estimated glomerular filtration rate
CKD-EPI: Chronic Kidney Disease Epidemiology Collaboration
MET: Metabolic equivalent of task
MICE: Multiple imputation by chained equations
MEC: Mobile examination center
NHANES: National Health and Nutrition Examination Survey
NSF: National Sleep Foundation
PHQ-9: 9-item Patient Health Questionnaire
RRR: Relative risk ratio
US: United States
WHO: World Health Organization
