# Supplementary material for: Factors associated with habitual sleep duration in US adults with hypertension: a cross-sectional study of the 2015–2018 National Health and Nutrition Examination Survey
Source: BMC Public Health. 2022 Jan 6;22:43. doi: 10.1186/s12889-021-12465-2 (PMC8739698; doi:10.1186/s12889-021-12465-2)
Supplement: Supplementary file 1 — Additional file 1: Supplemental Table 1. Comparative sensitivity analysis between pre-imputation and post-imputation data of variables with missing data. [file 12889_2021_12465_MOESM1_ESM.docx]

**Factors Associated with Habitual Sleep Duration Among US Adults with Hypertension – Supplementary Tables**

**Supplemental Table 1. Comparison of the distribution of pre-imputation and post-imputation data of variables with missing data.**

|  | **Pre-imputation** | **Post-imputation** |
| --- | --- | --- |
| **Variable** | **Weighted % (95% CI)** | **Weighted % (95% CI)** |
| **Marital status** |  |  |
| Married/Living with partner | 63.1 (60.4 – 65.8) | 63.1 (60.4 – 65.8) |
| Unmarried | 36.9 (34.2 – 39.6) | 36.9 (34.2 – 39.6) |
| **Education Level** |  |  |
| College graduate | 26.9 (23.5 – 30.6) | 26.9 (23.3 – 30.5) |
| Some college | 33.2 (30.8 – 35.7) | 33.2 (30.7 – 35.7) |
| High school graduate | 26.0 (23.8 – 28.3) | 26.0 (23.7 – 28.2) |
| Less than high school | 13.9 (12.2 – 15.9) | 13.9 (12.1 – 15.8) |
| **Income to Poverty Ratio category** |  |  |
| ≥4.00 | 36.6 (32.7 – 40.6) | 35.6 (31.7 – 39.4) |
| 2.00 - 3.99 | 29.8 (27.6 – 32.1) | 29.7 (27.5 – 31.9) |
| 1.00 - 1.99 | 21.1 (19.1 – 23.3) | 21.7 (19.6 – 23.8) |
| <1.00 | 12.5 (10.5 – 14.7) | 13.1 (11.0 – 15.2) |
| **Has health insurance** |  |  |
| Yes | 89.8 (87.3 – 91.9) | 89.8 (87.5 – 92.1) |
| No | 10.2 (8.1 – 12.7) | 10.2 (7.9– 12.5) |
| **Depressive symptoms severity** |  |  |
| Minimal or none | 74.7 (72.8 – 76.4) | 74.7 (72.9 – 76.5) |
| Mild | 16.5 (15.1 – 18.1) | 16.5 (15.0 – 18.1) |
| Moderate | 5.8 (5.0 – 6.7) | 5.8 (4.9 – 6.6) |
| Moderately severe to severe | 3.0 (2.4 – 3.8) | 3.0 (2.4 – 3.7) |
| **BMI (kg/m^2^)** |  |  |
| <25 | 18.1 (16.3 – 20.1) | 18.1 (16.3 – 20.0) |
| 25 - <30 | 30.5 (28.7 – 32.5) | 30.5 (28.6 – 32.4) |
| 30 - <35 | 25.7 (24.0 – 27.5) | 25.7 (24.0 – 27.5) |
| 35 - <40 | 13.8 (12.3 – 15.5) | 13.8 (12.2 – 15.4) |
| ≥40 | 11.8 (10.3– 13.4) | 11.8 (10.3 – 13.3) |
| **Cigarette smoking** |  |  |
| Never smoker | 52.8 (50.5 – 55.1) | 52.8 (50.5 – 55.1) |
| Current smoker | 17.2 (15.5 – 18.9) | 17.2 (15.5 – 18.8) |
| Former smoker | 30.1 (28.0 – 32.2) | 30.1 (28.0 – 32.2) |
| **Alcohol intake** |  |  |
| None | 29.4 (27.2 – 31.6) | 29.6 (27.5 – 31.8) |
| Moderate | 38.6 (35.9 – 41.4) | 38.4 (35.7 – 41.0) |
| Heavy | 32.0 (30.0 – 34.1) | 32.0 (30.0 – 34.0) |

*Abbreviations: CI, confidence interval; COPD, chronic obstructive pulmonary disease; BMI, body mass index*
