# Supplementary material for: Factors associated with habitual sleep duration in US adults with hypertension: a cross-sectional study of the 2015–2018 National Health and Nutrition Examination Survey
Source: BMC Public Health. 2022 Jan 6;22:43. doi: 10.1186/s12889-021-12465-2 (PMC8739698; doi:10.1186/s12889-021-12465-2)
Supplement: Supplementary file 2 — Additional file 2: Supplemental Table 2. Comparison of results on factors associated with short and long sleep duration – complete case analysis and post-imputation. [file 12889_2021_12465_MOESM2_ESM.docx]

**Factors Associated with Habitual Sleep Duration Among US Adults with Hypertension**

**Supplemental Table 2. Comparison of results on factors associated with short and long sleep duration – complete case analysis and post-imputation ^a^**

|  | **Short (vs adequate) sleep duration** | | **Long (vs adequate) sleep duration** | |
| --- | --- | --- | --- | --- |
|  | **Pre-imputation** | **Post-imputation** | **Pre-imputation** | **Post-imputation** |
| **Variable** | **aRRR (95% CI)** | **aRRR (95% CI)** | **aRRR (95% CI)** | **aRRR (95% CI)** |
| **Age** |  |  |  |  |
| 18-44 | REF | REF | REF | REF |
| 45 - 64 | 1.18 (0.89, 1.56) | 1.20 (0.96, 1.51) | 0.75 (0.44 1.28) | 0.72 (0.46-1.13) |
| 65 and above | 0.63 (0.42, 0.94)* | 0.63 (0.45-0.91)* | 0.67 (0.43, 1.04) | 0.74 (0.50-1.09) |
| **Female** | 0.65 (0.50 0.84)** | 0.70 (0.56, 0.88)** | 1.35 (1.04, 1.74)* | 1.24 (1.001-1.54)* |
| **Marital status** |  |  |  |  |
| Married/Living with partner | REF | REF | REF | REF |
| Unmarried | 1.17 (0.95, 1.43) | 1.14 (0.95, 1.37) | 1.13 (0.79, 1.62) | 1.06 (0.78-1.45) |
| **Race/Ethnicity** |  |  |  |  |
| Non-Hispanic White | REF | REF | REF | REF |
| Non-Hispanic Black | 2.18 (1.68, 2.82)*** | 2.08 (1.61, 2.67)*** | 1.03 (0.71, 1.48) | 1.13 (0.81-1.57) |
| Hispanic | 1.10 (0.78, 1.55) | 1.17 (0.82, 1.68) | 0.90 (0.62, 1.30) | 1.08 (0.80-1.46) |
| Non-Hispanic Asian | 1.34 (0.89, 2.02) | 1.23 (0.79, 1.91) | 1.03 (0.56, 1.89) | 1.12 (0.68-1.84) |
| Other | 1.01 (0.64, 1.62) | 1.22 (0.76, 1.96) | 0.60 (0.33, 1.10) | 0.78 (0.48-1.28) |
| **Country of Birth** |  |  |  |  |
| US-born | REF | REF | REF | REF |
| Not US-born | 1.01 (0.71, 1.44 | 1.16 (0.83-1.63) | 0.76 (0.44, 1.32) | 0.71 (0.47-1.07) |
| **Education Level** |  |  |  |  |
| College graduate | REF | REF | REF | REF |
| Some college | 1.28 (0.95, 1.72) | 1.44 (0.92-1.68) | 0.75 (0.44, 1.30) | 0.85 (0.51-1.43) |
| High school graduate | 1.28 (0.94, 1.75) | 1.17 (0.85-1.60) | 1.37 (0.74, 2.51) | 1.42 (0.86-2.34) |
| Less than high school | 1.38 (0.97, 1.95) | 1.21 (0.88-1.67) | 1.70 (0.87, 3.32) | 1.59 (0.88-2.89) |
| **Income to Poverty Ratio** |  |  |  |  |
| ≥4.00 | REF | REF | REF | REF |
| 2.00 - 3.99 | 1.23 (0.96, 1.56) | 1.21 (0.95-1.56) | 1.26 (0.74, 2.13) | 1.20 (0.74-1.95) |
| 1.00 - 1.99 | 0.91 (0.65, 1.27) | 0.94 (0.69-1.28) | 1.40 (0.77, 2.51) | 1.28 (0.77-2.14) |
| <1.00 | 0.96 (0.65, 1.42) | 0.97 (0.67-1.39) | 1.60 (0.86, 2.98) | 1.45 (0.82-2.58) |
| **Employment Status** |  |  |  |  |
| Works 35 – 44 hours/week | REF | REF | REF | REF |
| Works <35 hours/week | 0.85 (0.52, 1.38) | 0.88 (0.61-1.28) | 1.83 (1.09, 3.07)* | 1.71 (1.07-2.73)* |
| Works ≥45 hours/week | 1.86 (1.25, 2.77)** | 1.81 (1.32-2.48)** | 0.67 (0.33, 1.35) | 0.66 (0.34-1.29) |
| Not working – health reasons | 1.18 (0.74, 1.90) | 1.12 (0.77-1.63) | 4.62 (2.72, 7.85)*** | 4.87 (2.89-8.22)*** |
| Not working - retired | 1.39 (0.94, 2.06) | 1.41 (1.01-1.98) * | 3.58 (2.18, 5.88)*** | 3.46 (2.18-5.49)*** |
| Not working – other reasons | 0.86 (0.58, 1.26) | 0.85 (0.65-1.12) | 3.30 (1.73, 6.29)** | 3.29 (1.84-5.88)*** |
| **No health insurance** | 1.29 (0.95, 1.76) | 1.30 (0.97-1.75) | 1.06 (0.73, 1.55) | 1.09 (0.75-1.59 |
| **Comorbidities** |  |  |  |  |
| Heart disease | 1.32 (0.91, 1.93) | 1.20 (0.80-1.78) | 1.01 (0.66, 1.53) | 0.88 (0.59-1.30) |
| Stroke | 0.84 (0.58, 1.21) | 0.79 (0.54-1.16) | 0.90 (0.58, 1.40) | 1.09 (0.78-1.54) |
| COPD or current asthma | 1.11 (0.81, 1.53) | 1.10 (0.83-1.47) | 1.01 (0.74, 1.39) | 0.98 (0.72-1.35) |
| Arthritis | 0.98 (0.79 1.23) | 1.06 (0.89-1.26) | 0.86 (0.64, 1.17) | 0.93 (0.71-1.21) |
| Diabetes mellitus | 1.12 (0.84, 1.50) | 1.08 (0.83-1.40) | 1.44 (0.99, 2.09) | 1.37 (0.99-1.88) |
| Chronic kidney disease | 1.29 (0.90 1.85) | 1.18 (0.86-1.61) | 1.44 (1.09, 1.92)* | 1.48 (1.14-1.92)* |
| **Depressive symptoms** |  |  |  |  |
| Minimal or none | REF | REF | REF | REF |
| Mild | 1.08 (0.83, 1.41) | 1.12 (0.88-1.44) | 1.32 (0.91, 1.91) | 1.35 (0.95-1.93) |
| Moderate | 1.87 (1.23, 2.85)** | 1.61 (0.99-2.61) | 1.52 (0.98, 2.37) | 1.62 (1.08-2.44) * |
| Moderately severe to severe | 1.87 (0.91, 3.85) | 1.66 (0.88-3.14) | 1.78 (0.84, 3.76) | 1.89 (1.05-3.43) * |
| **Help-seeking for sleeping difficulty** | 1.20 (0.97, 1.50) | 1.25 (1.02-1.53)* | 0.83 (0.58, 1.20) | 0.84 (0.61-1.16) |
| **BMI category** |  |  |  |  |
| <25 | REF | REF | REF | REF |
| 25 - <30 | 1.24 (0.87, 1.76) | 0.98 (0.70-1.37) | 1.12 (0.74, 1.71) | 1.00 (0.72-1.39) |
| 30 - <35 | 1.07 (0.76, 1.50) | 0.93 (0.65-1.34) | 1.08 (0.64, 1.82) | 1.06 (0.69-1.62) |
| 35 - <40 | 1.16 (0.80, 1.68) | 1.02 (0.70-1.48) | 0.71 (0.38, 1.34) | 0.71 (0.42-1.22) |
| ≥40 | 1.34 (0.95, 1.88) | 1.19 (0.85-1.66) | 0.93 (0.53, 1.63) | 0.91 (0.56-1.45) |
| **Alcohol intake** |  |  |  |  |
| None | REF | REF | REF | REF |
| Moderate | 0.83 (0.65, 1.06) | 0.84 (0.67-1.05) | 1.02 (0.74, 1.40) | 0.99 (0.76-1.30) |
| Heavy | 0.91 (0.67, 1.23) | 0.89 (0.68-1.17) | 0.94 (0.64, 1.38) | 0.91 (0.64-1.31) |
| **Cigarette smoking** |  |  |  |  |
| Never smoker | REF | REF | REF | REF |
| Current smoker | 1.19 (0.86, 1.65) | 1.25 (0.93-1.68) | 1.26 (0.82, 1.92) | 1.16 (0.82-1.63) |
| Former smoker | 0.85 (0.68, 1.08) | 0.88 (0.72-1.07) | 1.05 (0.79, 1.39) | 0.96 (0.72-1.29) |
| **Physical activity level** |  |  |  |  |
| Sufficient | REF | REF | REF | REF |
| None | 1.10 (0.83, 1.45) | 1.18 (0.89-1.57) | 1.16 (0.71, 1.90) | 1.39 (0.90-2.13) |
| Low | 0.78 (0.55, 1.10) | 0.82 (0.59-1.15) | 0.76 (0.49, 1.18) | 0.91 (0.59-1.40) |
| High | 1.06 (0.76, 1.48) | 1.14 (0.85-1.54) | 1.01 (0.61, 1.67) | 1.05 (0.65-1.71) |

***^a^*** *All covariates adjusted for all other covariates in the model*

*Complete case analysis, n=4,520. Multiple imputation: n=5660*

* *P* *value* < 0.05; ** *P value* < 0.01; ****P value* < 0.001

*Abbreviations: aRRR, adjusted relative risk ratio; CI, confidence interval*

*The results highlighted in yellow are the ones that showed differences between complete case analysis and imputed data analyses*
